# Yearly hypertension screening in women with a history of pre-eclampsia: a cost-effectiveness analysis

**DOI:** 10.1007/s12471-015-0760-z

**Published:** 2015-10-08

**Authors:** J.T. Drost, J.P.C. Grutters, G.-J. van der Wilt, Y.T. van der Schouw, A.H.E.M. Maas

**Affiliations:** 1 Department of Cardiology, Isala Klinieken, 8000 PO Box 10500, GM Zwolle, the Netherlands; 2 Department for Health Evidence, Radboud University Medical Center, Nijmegen, the Netherlands; 3 Julius Center for Health Sciences and Primary Care, University Medical Center Utrecht, Utrecht, the Netherlands; 4 Department of Cardiology, Radboud University Medical Center, Nijmegen, the Netherlands

**Keywords:** Preeclampsia, Economic analysis, Cardiovascular screening

## Abstract

**Background:**

Women with a history of preeclampsia are at increased risk for future hypertension and cardiovascular disease (CVD); until now it is not clear whether preventive measures are needed.

**Methods:**

A decision-analytic Markov model was constructed to evaluate healthcare costs and effects of screening and treatment (100 % compliance) for hypertension post preeclampsia based on the available literature. Cardiovascular events and CVD mortality were defined as health states. Outcomes were measured in absolute costs, events, life-years and quality-adjusted life-years (QALYs). Sensitivity and threshold analyses were performed to address uncertainty.

**Results:**

Over a 20-year time horizon, events occurred in 7.2 % of the population after screening, and in 8.5 % of the population without screening. QALYs increased from 16.37 (no screening strategy) to 16.40 (screening strategy), an increment of 0.03 (95 % CI 0.01;0.05) QALYs. Total expected costs were € 8016 in the screening strategy, and € 9087 in the none screening strategy (expected saving of € 1071 (95 % CI − 3146;-87) per person).

**Conclusion:**

Annual hypertension screening and treatment in women with a history of preeclampsia may save costs, for at least a similar quality of life and survival due to prevented CVD compared with standard care.

**Electronic supplementary material:**

The online version of this article (doi:10.1007/s12471-015-0760-z) contains supplementary material, which is available to authorized users.

## Introduction

It has been well established that women with a history of preeclampsia are at increased risk of future cardiovascular disease (CVD) morbidity and mortality [1]. Preeclampsia is defined as de novo hypertension (140/90 mmHg) with proteinuria (> 0.3 g/24 h) occurring in the second half of pregnancy [2]. Despite the abundant evidence on the increased CVD risk in these women later in life, [3, 4] intermediate follow-up data are still relatively scarce and it is still undefined whether preventive measures are needed [5].

In the Preeclampsia Risk EValuation in FEMales (PREVFEM) cohort, consisting of 339 women with a history of early preeclampsia (before 32 weeks of pregnancy), we previously identified hypertension as the most important CVD risk factor at a cardiovascular screening 10 years post-partum [6]. As hypertension is an established CVD risk factor [7], early detection and treatment of hypertension is of primary importance for this category of young women. However, screening onwards from pregnancy is labour-intensive and may be costly. Currently, few data on cost-effectiveness of preventive interventions in women after preeclampsia are available; however, data on women after early preeclampsia are not available [8]. We performed a model-based cost-effectiveness analysis to estimate the healthcare costs and potential effects of screening for hypertension in women with a history of early preeclampsia.

## Methods

### Overview

**Fig. 1:**
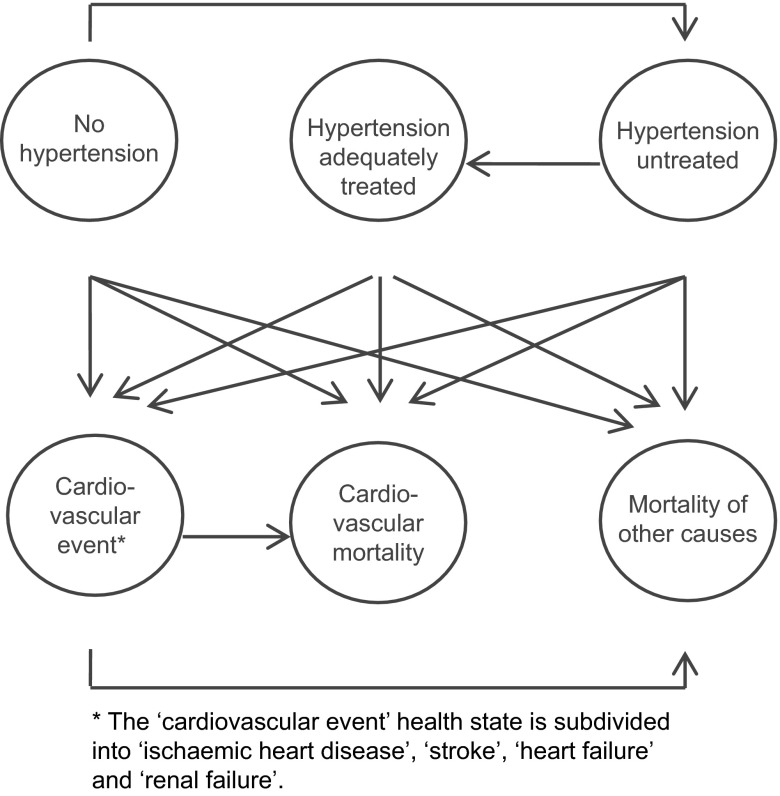
Schematic representation of the Markov model.

A decision-analytic Markov model was constructed to evaluate costs and effects of screening for hypertension from a healthcare perspective in women post preeclampsia. In each cycle of the model patients were transferred to a certain health state according to the further described transition probabilities. The predefined health states are demonstrated in Fig. 1. The cycle length was one year. We used time horizons of 10 and 20 years, the starting point was at 30 years of age. Outcomes were measured in number of events, life-years, quality-adjusted life-years (QALYs) and absolute costs. The Markov model was built and analysed in Microsoft Office Excel 2010.

### Model construction

#### Patient characteristics

As target population we used the PREVFEM cohort, comprising women with a history of early-onset preeclampsia (onset before 32 weeks of pregnancy).[6] This study was performed to evaluate the presence of CVD risk factors in women at 10 years after index pregnancy. The detailed protocol of the screening procedure has been described elsewhere.[6]

#### Comparators

We introduced a hypothetical annual blood pressure screening at the general practitioner (GP) for women after preeclampsia, starting in the first-year postpartum. The comparative strategy existed of care as usual: standard obstetric care and no specifically arranged blood pressure check-ups. If hypertension was detected in any of the strategies, this involved three additional GP visits, an ECG recording and prescription of medication. The probability of developing CVD with and the probability without hypertension were equal between the two strategies. The only difference between the strategies was in detecting and therefore treating hypertension.

#### Transition probabilities

Risk of hypertension and presence of adequate treatment for women after preeclampsia were based on the PREVFEM cohort [6]. As the follow up in this cohort is relatively short (10 years) we used published literature to assess the risk of future CVD in women post preeclampsia (early as well as late preeclampsia) (Supplementary data, Table 1) [3, 9, 10].

Probabilities of developing CVD for each cycle (Table 1) were derived from meta-analyses on cardiovascular disease [3, 9, 10]. Risk reduction in the intervention group for the development of ischaemic heart disease and stroke were based on the effects of blood pressure lowering, starting with a pre-treatment systolic blood pressure (SBP) of 140 mmHg and one drug standard dose [11]. As Law et al. did not describe the effects of blood pressure lowering on heart failure and mortality we used hazard ratios (per 20 mmHg SBP increase) on CVD mortality and total CVD events in a Dutch cohort study (35–65 years of age) to estimate the effects on these health states [12]. Estimation of the relative risk for the development of end-stage renal disease was also based on an SBP difference of 20 mmHg in a American cohort of 37 years of age [13].

**Table 1:** Relative risk on cardiovascular outcome in participants with untreated hypertension

Health outcome	Relative risk (95 % CI)	Distribution	Reference
Ischaemic heart disease	1.32	Fixed	11
Stroke	1.49	Fixed	11
Heart failure	1.47 (1.35–1.61)	Normal	12
End-stage renal disease	2.57 (2.06–3.22)	Normal	13
Cardiovascular mortality	1.61 (1.35–1.92)	Normal	12

#### Costs

Costs (Supplementary data, Table 2) were presented in Euros (€). Price indices were used to convert costs to the 2012 price level, future expenditures were discounted to their present value by a rate of 4 % [14]. Estimation of costs in the intervention strategy was based on a single screening visit at the GP of € 30 (Dutch reference price, as established by the Dutch Healthcare Insurance Board) [14]. Costs for detection of hypertension in both strategies were based on three GP visits and an ECG recording (€ 130) and yearly costs for treatment of hypertension and medication use according to current Dutch GP guidelines [15, 16]. Costs associated with the potential CVD and yearly ongoing costs thereafter were derived from published Dutch cost studies; [17, 18] if not available other European studies were used [19].

#### Effects

QALYs were used as outcome measure in the model. To measure health-related quality of life we used a single index utility, on a scale from 0 (death) to 1 (perfect health). Utility data (Supplementary data, Table 3) were derived from recent population-based literature [20, 24]. Because of the general preference to enjoy benefits as soon as possible, future effects were discounted to their present value by a rate of 1.5 % [14].

### Analysis

Total event rates, life-years, QALYs and expected costs were calculated for both strategies. If one strategy was more effective and less costly, this strategy was deemed cost-effective. If screening was more costly and more effective, or the opposite, incremental cost-effectiveness ratios (ICERs) were calculated by dividing the incremental costs by the incremental QALYs. Whether screening is cost-effective depends on whether this ICER is below the societal willingness to pay for a QALY. The informal willingness to pay in the Netherlands is € 20,000 per QALY for standard health interventions [14].

#### Sensitivity analysis

To reflect the uncertainty of the parameter estimates in the model probabilistic sensitivity analyses were performed [25]. For this purpose distributions were assigned to the model parameters. Parameter values were drawn at random from the assigned distributions, using Monte Carlo simulation with 5000 iterations. Based on these simulations, mean values and 2.5th and 97.5th percentiles (95 % credible intervals) surrounding the costs and effects were calculated.

The results of this probabilistic analysis are demonstrated in a cost-effectiveness plane and cost-effectiveness acceptability curve (CEAC). The CEAC shows the probability that screening is cost-effective over a range of willingness-to-pay thresholds [26].

In addition, a threshold analysis was performed to determine the maximum costs of screening for which screening is the most effective and least expensive strategy.

One-way deterministic sensitivity analyses were performed to evaluate the effect of reduced adherence to hypertension treatment in women (75 %), as in common clinic practice adherence to antihypertensive medication is less than 100 %. Deterministic sensitivity analyses were also used to evaluate changes in quality of life of women with hypertension. In our primary model we set utility index for women with and without hypertension at 0.98 [20], we propose that hypertension and use of medication might decrease quality of life.

## Results

### Base-case analysis

Screening for hypertension was found to be slightly more effective than no screening (Table 2). Over a time horizon of 20 years events occurred in 7.2 % of the population after screening, and in 8.5 % of the population without screening. Life-years in the screening strategy were 16.9529 versus 16.9427 per person in the no screening strategy, an increment of 0.0102 (95 % CI 0.0053; 0.0158) life-years. QALYs increased to 16.37 for the no screening strategy and to 16.40 for the screening strategy, an increment of 0.0320 (95 % CI 0.0143; 0.0533) QALYs, which equals 12 days in perfect health quality.

**Table 2:** Cost-effectiveness results (probabilistic analysis)

	Screening strategy Mean (95 % credible intervals)	No screening strategy Mean (95 % CI)	Increment Mean (95 % CI)
Expected health care costs (€/horizon)			
10 year horizon	3058 (1893; 5446)	3083 (1755; 5778)	− 25 (− 392; 142)
20 year horizon	8016 (4614; 14627)	9087 (4721; 17,785)	− 1071 (− 3146; − 87)
Expected life years (years/horizon)	9.1716	9.1701	0.0015
10 year horizon	(9.1676; 9.1752) 16.9529	(9.1659; 9.1741) 16.9427	(0.0007 0.0022) 0.0102
20 year horizon	(16.9390; 16.9660)	(16.9259; 16.9583)	(0.0053; 0.0158)
Expected QALYs (QALYs/horizon) 10 year horizon 20 year horizon	8.93 (8.81; 9.03) 16.40 (16.15; 16.65)	8.92 (8.81; 9.02) 16.37 (16.11; 16.63)	0.0046 (0.0020;0.0078) 0.0320 (0.0143; 0.0533)

Screening was also found to be less expensive than no screening. Estimated expenditures over a 20-year time horizon in the screening strategy were € 8016, compared with €9087 in the no screening strategy. This results in further savings of €1071 (95 % CI − 3146; − 87) per person.

### Sensitivity analysis

**Fig. 2:**
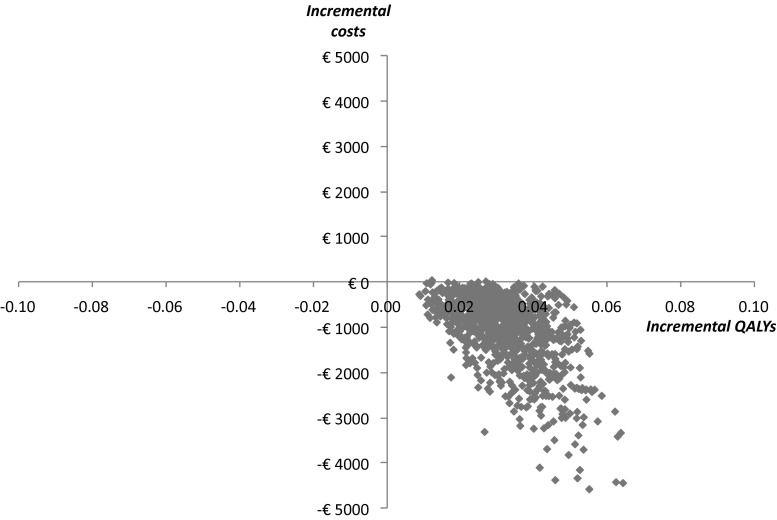
Effectiveness of screening for hypertension post preeclampsia according to the probabilistic analysis in 20 year time horizon

**Fig. 3:**
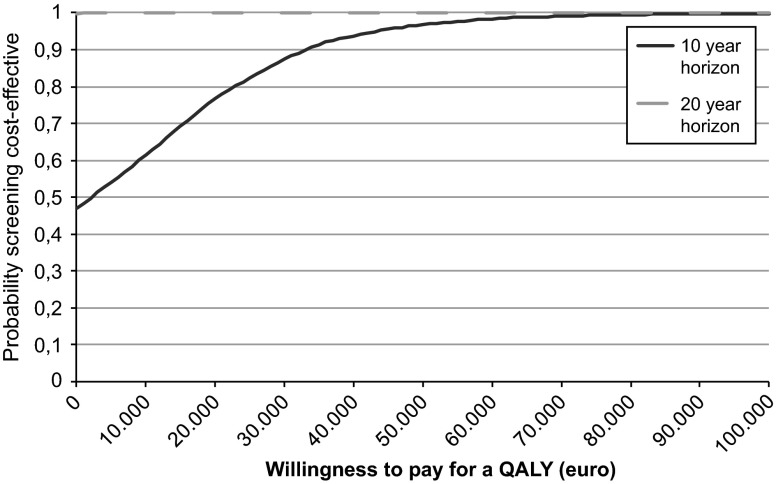
Cost-effectiveness acceptability curves for hypertension screenig in women post preeclampsia, combined results for 10 and 20 year time horizon

The results of the probabilistic sensitivity analysis showed that despite considerable uncertainty, in almost all simulations the screening strategy was less costly and more effective than the no screening strategy over 20 years (Fig. 2). From the CEAC it is clear that regardless of the willingness to pay for a QALY, screening has a probability of being cost-effective of over 99 % in 20 years (Fig. 3).

In the threshold analysis, screening remained the least costly strategy over 20 years with costs of screening up to € 151 per year.

Table 3 shows the one-way sensitivity analysis on adherence and utility. With an adherence percentage of 75 % cost savings decrease to € 1024 and the QALY gain to 0.031, but screening remains cost-effective. For each variation in utility the screening strategy remained the most effective strategy.

**Table 3:** Results of the deterministic threshold and sensitivity analysis (over 20 years)

	Incremental costs screening (euro/time horizon 20 y)	Incremental QALYs screening (QALYs/time horizon 20 y)	Incremental costs per QALY gained
Base case analysis	*–* 1062	0.032	Screening dominates
Costs of screening € 151/y	− 4	0.032	Screening dominates
Adherence on medication 75 %	− 1024	0.031	Screening dominates
Utility hypertension 0.95	− 1062	0.030	Screening dominates
Utility hypertension 0.90	− 1062	0.026	Screening dominates
Utility untreated hypertension 0.95	− 1062	0.184	Screening dominates
Utility untreated hypertension 0.90	− 1062	0.437	Screening dominates
Utility untreated hypertension 0.90, treated hypertension 0.95	− 1062	0.283	Screening dominates
Utility untreated hypertension 0.95, treated hypertension 0.97	− 1062	0.133	Screening dominates

## Discussion

Several studies have clearly demonstrated the increased risk for CVD in women with a history of preeclampsia [1, 3]. Especially women after early and severe preeclampsia are at higher risk for future CVD [1, 10]. The 2011 American Heart Association guidelines on cardiovascular disease prevention in women recognise pregnancy as a unique chance to predict women’s lifetime cardiovascular risk, as pregnancy-related complications may unmask premature vascular or metabolic diseases. Therefore, appropriate monitoring of CVD risk factors in these high-risk women is recommended [5]. The 2012 European Society of Cardiology guideline on CVD prevention also indicates that prevention of CVD in women ideally starts during pregnancy and lasts until end of life [7]. However, in standard primary care obstetric history is not yet routinely incorporated in risk assessment [27].

In this model-based analysis we assess the expected cost-effectiveness of a hypertension screening strategy in women after preeclampsia. We found that a relatively simple preventive strategy, consisting of a yearly blood pressure measurement at the GP’s surgery after index pregnancy, gives an expected saving of € 1071 per person and a slight increment of 0.03 QALY (12 days living in perfect health) per screened person.

### Strengths and limitations

The power of this Markov model is founded in the combination of the available evidence on CVD post preeclampsia. Our model could be of help in making evidence-based decisions on prevention post preeclampsia, without performing costly long-term follow-up studies.

However, our model has some limitations. To estimate risk of hypertension in women after preeclampsia, we used a cohort of women post early preeclampsia. As early preeclampsia confers the highest future CVD risk of all hypertensive pregnancy disorders [1, 10], the results of our Markov model should not be extrapolated to all women post hypertensive pregnancy disorders. As future risk for hypertension and CVD are lower in women with late compared with early preeclampsia, the magnitude of screening effects will be smaller.

Secondly, as prospective data in women after early preeclampsia are relatively scarce, we also used CVD risk data of patients with less severe preeclampsia to estimate risk of future CVD after preeclampsia. This may have underestimated the net effect of hypertension screening on CVD risk in women post early preeclampsia in our model. Nonetheless, the presented data are convincing and underline the need for preventive measures in women post early preeclampsia.

In our model we used mainly Dutch costs. As costs in other jurisdictions will be different from the Dutch situation, our results could be less generalisable to other health systems. However, in general costs in the US tend to be higher than in the Dutch healthcare system, so savings in the US will probably be larger. On the other hand, the Dutch well-structured GP system can adopt our intervention strategy easily, whereas this might be more difficult in other countries.

To perform our model we made some assumptions. First, we assumed that women with non-treated hypertension in the non-intervention strategy had a 20 mmHg higher SBP than women with adequately treated hypertension in the intervention strategy, based on our PREVFEM data. This seems reliable if women in the intervention strategy are directly well controlled and treated, but in current daily practice this is disappointing 28 s, in our model participants were all compliant in taking their antihypertensive medication, in real practice a quarter of women on blood pressure medication do not take their medication regularly, which will reduce the calculated preventive effects [29]. However, sensitivity analysis shows that screening remains cost-effective with an adherence rate of 75 %.

To estimate the increase in CVD risk in the presence of increased blood pressure we used a meta-analysis and two cohort studies; these studies consisted of men and women with an age distribution between 35 and 65 years. Use of data based on both men and women might have overrated over effects as men have higher CVD rates at younger ages [30].

### Clinical implications and future perspective

In our model we demonstrated that a yearly blood pressure measurement at the GP’s office in women after early preeclampsia is cost-effective in preventing CVD. However, in a time horizon of 20 years we found only a slight gain in QALYs (12 days living in perfect health). Although this gain is associated with cost savings, the clinical relevance of this small gain in QALYs can be debated. Besides this, we also need to consider the willingness of young, healthy women to use blood pressure medication, with potential side effects, for a 12-day longer life in perfect health. Also, it is unclear what the effects of routine screening are on quality of life in healthy young women. We expect a small negative effect on quality of life; however, because of a lack of data on the magnitude of these effects this was not incorporated in our model. A pilot study on screening might give more insight into these effects.

Further, before considering incorporation of a screening strategy into new primary care guidelines, it should be investigated if the strategy used is attainable for GPs. The potential burden for GPs may be decreased by starting screening at 10-year postpartum or by reducing the frequency of the screening. More research is needed to establish the best time schedule for hypertension screening. For the future, there might be an interesting role for e-health medicine in prevention of CVD in young high-risk women after preeclampsia.

## Conclusion

The presented Markov model demonstrates that annual hypertension screening and treatment in primary care in women after early preeclampsia might be cost-effective in preventing future CVD. However, the described assumptions, mainly regarding compliance and limited clinical effects, should be taken into account before any recommendations regarding screening programs post preeclampsia can be made.

## Electronic supplementary material

(DOCX 18 kb)
