# Supplementary material for: Yearly hypertension screening in women with a history of pre-eclampsia: a cost-effectiveness analysis
Source: Neth Heart J. 2015 Oct 8;23(12):585–91. doi: 10.1007/s12471-015-0760-z (PMC4651960; doi:10.1007/s12471-015-0760-z)
Supplement: Supplementary file 1 — (DOCX 18 kb) [file 12471_2015_760_MOESM1_ESM.docx]

**Table 1: Risk on cardiovascular outcome in women with a history of preeclampsia**

| **Health outcome** | **Risk (%)**  **(events/ total N)** | **Follow-up**  **(years)** | **Yearly probability** | **Reference** |
| --- | --- | --- | --- | --- |
| Hypertension (screening-strategy) | 43.1 (146/339) | 10 years | 0.0548 | 6 |
| Hypertension  (non-intervention strategy)   - On medication - Adequately treated | 20.6 (70/339)  8.0 (27/339) | 10 years  10 years |  | 6 |
| Ischaemic heart disease | 1.8 (458/25,148) | 14.6 years | 0.0012 | 10 |
| Stroke | 1.6 (412/25,184) | 14.6 years | 0.0011 | 10 |
| Heart failure | 0.5 (123/25,184) | 14.6 years | 0.0003 | 10 |
| End-stage renal disease | 0.8 (2/242) | 40 years | 0.0006 | 9 |
| Cardiovascular mortality | 0.6 (167/26,168) | 10-42 years | 0.0005 | 3 |

**Table 2: Healthcare costs**

| **Health outcome** | **Event related costs (€)** | **Long-term treatment costs / year (€)** | **Distribution** | **Reference** |
| --- | --- | --- | --- | --- |
| Hypertension   - Screening/ year - Treatment | 29.73  129.52 | -  34.89 | Fixed  Fixed | 15,16 |
| Ischaemic heart disease | 16,570 | 1007 | Fixed | 14 |
| (Major) stroke | 34,585 | 20,194 | Fixed | 14 |
| Heart failure | 3707 | 1550 | Fixed | 17, 18 |
| End-stage renal disease | - | 53,961 | Fixed | 19 |
| Cardiovascular mortality | 4000 | - | Fixed | 18 |

**Table 3: Health outcome associated utility**

| **Health outcome** | **Utility (SD)** | **Distribution** | **Reference** |
| --- | --- | --- | --- |
| No hypertension | As hypertension |  |  |
| Hypertension | 0.98 (0.005) | Beta | 20 |
| Ischaemic heart disease | 0.837 (0.17) | Beta | 21 |
| Stroke | 0.70 (0.27) | Beta | 22 |
| Heart failure | 0.47 (0.32) | Beta | 23 |
| End-stage renal disease | 0.71 (0.05) | Beta | 24 |
